# Autism spectrum disorder, flea and tick medication, and adjustments for exposure misclassification: the CHARGE (CHildhood Autism Risks from Genetics and Environment) case–control study

**DOI:** 10.1186/1476-069X-13-3

**Published:** 2014-01-23

**Authors:** Alexander P Keil, Julie L Daniels, Irva Hertz-Picciotto

**Affiliations:** 1 Department of Epidemiology, CB 7435, University of North Carolina, Chapel Hill, NC 27599-7435, USA; 2 School of Medicine and the MIND (Medical Investigations of Neurodevelopmental Disorders) Institute, University of California Davis MS1C, Davis, CA 95616, USA

**Keywords:** Autism, Bayesian, Imidacloprid, Measurement error, Neonicotinoids, Pesticides

## Abstract

**Background:**

The environmental contribution to autism spectrum disorders (ASD) is largely unknown, but household pesticides are receiving increased attention. We examined associations between ASD and maternally-reported use of imidacloprid, a common flea and tick treatment for pets.

**Methods:**

Bayesian logistic models were used to estimate the association between ASD and imidacloprid and to correct for potential differential exposure misclassification due to recall in a case control study of ASD.

**Results:**

Our analytic dataset included complete information for 262 typically developing controls and 407 children with ASD. Compared with exposure among controls, the odds of prenatal imidacloprid exposure among children with ASD were slightly higher, with an odds ratio (OR) of 1.3 (95% Credible Interval [CrI] 0.78, 2.2). A susceptibility window analysis yielded higher ORs for exposures during pregnancy than for early life exposures, whereas limiting to frequent users of imidacloprid, the OR increased to 2.0 (95% CI 1.0, 3.9).

**Conclusions:**

Within plausible estimates of sensitivity and specificity, the association could result from exposure misclassification alone. The association between imidacloprid exposure and ASD warrants further investigation, and this work highlights the need for validation studies regarding prenatal exposures in ASD.

## Background

Autism spectrum disorders (ASD) are a set of developmental conditions characterized by a constellation of impairments in social interaction and communication, and repetitive patterns of interests or behaviors [1-4]. The etiology of ASD is largely unknown. Prenatal and early life exposures to pesticides have been of growing concern in relation to birth outcomes and children’s neurologic or neurocognitive development [5-13]. A few studies have described associations between ASD and pesticides [9,14,15].

Animal models have revealed that prenatal exposure to the insecticide imidacloprid induces neurobehavioral deficits [16,17]. Imidacloprid kills a variety of insects through action on the nicotinic receptor [18]. Imidacloprid is used agriculturally and it is an active ingredient of flea and tick treatments for household pets [19]. Imidacloprid was introduced for consumer use in 1999 and usage has rapidly increased since, but its health effects on humans are poorly understood [18,19]. The current analysis examines whether household imidacloprid usage as a flea and tick treatment on household pets is associated with ASD using data from a large case–control study with extensive, maternally-reported information on prenatal exposures.

Because investigation of this association is novel and imidacloprid exposure is characterized by parent report, we rigorously explore the potential for any observed associations to be obscured or inflated by systematic biases due to misclassification of imidacloprid exposure.

In case–control studies, use of retrospectively reported exposures can be subject recall bias, and overly broad exposure definitions can attenuate the apparent effects of etiologically relevant exposures [20]. We apply analytic methods to address both of these potential sources of error using frequentist and Bayesian methods to account for exposure measurement error and we perform several sensitivity analyses [21,22]. While correcting for exposure misclassification is possible using frequentist methods, Bayesian methods were preferable for several reasons. Practically speaking, our approach was a natural fit for a Bayesian framework in which we can fit a single (complex) model rather than piecing together several frequentist models. In principle, Bayesian methods also allow explicit inclusion of prior knowledge and can preclude time-consuming model selection procedures through stabilization, rather than elimination, of regression model terms [23].

The aims of the analysis are to 1) estimate associations between imidacloprid and ASD adjusted for confounders, but without accounting for exposure misclassification (frequentist and Bayesian), 2) utilize restriction and time-window analysis to estimate associations in which exposure measurement error may be less severe (frequentist) and 3) estimate the posterior OR for a range of potential exposure misclassification scenarios (Bayesian).

## Methods

### Participant recruitment

Participants were recruited as part of the ongoing Childhood Autism Risks from Genetics and Environment (CHARGE) Study, a population based, case–control investigation of the environmental and genetic causes of autism conducted at the UC Davis MIND (Medical Investigations of Neurodevelopmental Disorders) Institute. Children previously identified as having ASD are recruited from: an administrative database of the California Department of Developmental Services, which contract 21 Regional Centers to coordinate services for persons with developmental disabilities; health and service providers; other MIND Institute research studies; and self-referrals. Regional Centers are estimated to provide services to 75-80% of all children with ASD in their catchment areas [24]. General population controls were recruited from state birth records, with frequency matching to the age and Regional Center distribution and the projected sex distribution of the ASD cases. Further details of the CHARGE Study have been described elsewhere [25].

### Outcome

ASD was assessed using the Autism Diagnostic Interview-Revised and the Autism Diagnostic Observation Schedules [26,27]. Clinically trained study personnel determined cognitive function using the Mullen Scales of Early Learning and adaptive function using the Vineland Adaptive Behavior Scales and retrospectively assessed developmental trajectory in all children with the Child Development Questionnaire (a subset of the Early Development Questionnaire) [28-30]. The Social Communications Questionnaire (SCQ) was administered to general population controls and those who screened positive were assessed for autism spectrum disorders [31]. Study personnel assigned a final case status of ASD based on standard cut-offs for the Autism Diagnostic Interview-Revised and Autism Diagnostic Observation Schedules, (e.g. Risi et al. [32]) or typically developing based on an SCQ score <15 and scoring above the cut-off of two standard deviations below the mean on tests of cognitive and adaptive function.

### Exposure

Exposure and confounder data were collected through maternal phone interview from the Environmental Exposure Questionnaire. Interviewers were trained in this instrument and were available for English or Spanish speakers. Household imidacloprid usage was determined from the response to the question "During the index time until now, did you or anyone in your household use sprays, dusts, powders or skin applications for fleas or ticks on pets?" (‘index period’ was defined as 3 months prior to conception through breastfeeding). This was followed by identification of which product was used and when (which months during the period 3 months before conception to birth, and in which years after birth). Children were considered exposed if their mother reported use of Advantage and K9 Advantix on pets, which contain approximately 9% imidacloprid. The recommended use of the product is monthly application to pets. We classified exposure as consistent (use at least once each month during pregnancy) or occasional (used less than once each month during pregnancy). No other household products containing imidacloprid were reported to have been used [18]. We considered a child exposed prenatally (yes = 1 or no = 0) if the mother reported any household usage from 3 months before conception until birth. We also classified exposure as separate binary indicators for the three-month period prior to conception, each trimester of pregnancy, and each year of the child’s life, up to age two.

### Additional covariates

We created a Directed Acyclic Graph (DAG) to select *a priori* covariates for our statistical models based on existing literature and plausible relationships among them [33]. Hypothesized risk factors chosen for control were on unblocked backdoor paths and not downstream from exposure or outcome [34]. Covariates selected from our DAG were: Maternal education [high school, college degree (reference), and at least some college], race/ethnicity [White/non-Hispanic (reference), other], parity [ordinal integer] and pet ownership during pregnancy [1 = yes, 0 = no], as well as the matching factors (child’s sex [male = 0, female = 1] and age at interview [in years, centered at the mean], and region of birth [indicator variables, 5 categories]).

### Statistical analysis

#### Associations between imidacloprid and ASD

To address our primary aim, we used Bayesian methods for logistic regression [21]. Prior estimates for regression parameters (for maternal education, race, and parity) were derived from crude estimates provided in five studies on perinatal risk factors for ASD [35-39]. These studies were chosen because the study samples were similar to the CHARGE sample. Regression coefficients for which we could obtain no valid prior information (those for the effects of imidacloprid, the matching factors, pet ownership, and the intercept) were given normally distributed priors with a mean of 0 and a variance of 10 (N (0,10)). These regression coefficients are reported in detail in Table 1 of the Additional file 1. This analysis assumes perfect exposure classification, which we refer to as our “naïve” model.

To investigate whether the association differs by the reported timing of exposure, we estimated the OR in separate models for the three month pre-conception period, each trimester, and first three years of life. We performed this analysis using frequentist logistic regression models because we have little prior knowledge regarding how to set time-specific misclassification priors, since these are rarely, if ever, reported in relevant literature. We also report the results of models stratified by consistency of imidacloprid use (used throughout the entire prenatal period, or only used in part of the prenatal period).

#### A Bayesian approach to correcting for potential exposure misclassification

To correct for potential misreporting of exposure by mothers in CHARGE, we estimated the OR of imidacloprid exposure among children with ASD and typically developing children using a Bayesian approach described by Gustafson [21] and previously applied by MacLehose et al. [22]. This method uses 3 jointly estimated models to simultaneously model the “true” exposure and estimate its association with ASD. One model was used to model the probability of the “true” exposure as a function of all covariates (using N(0,1) prior estimates for model coefficients) in a logistic model. In the second model we parameterize the probability of “true” exposure, given reported exposure and case/control status, with prior parameter estimates of exposure misclassification (sensitivity and false-positive probability [i.e., 1-specificity]) described below. In a third model, the probability of a child in the study being diagnosed with ASD was modeled as a function of the “true” exposure and all covariates. These probabilities were then used to estimate the posterior OR of the ASD-imidacloprid association. As a sensitivity analysis, we examined the influence of our priors on regression parameters of the third model by examining posterior estimates derived using vague (~N (0,10), tight (~N (0,1)), and informative regression parameter priors.

Our model obtains the posterior estimates using Markov Chain Monte Carlo methods [40], which approximate the analytic solution to the joint-probability model for case–control data with misclassified exposures described by described by Gustafson [21]. Roughly, the intuition behind this approach is that we use information on case status (ASD or TD) and exposure probability to simulate our data under a scenario in which exposure is reported prospectively, rather than retrospectively (and thus is not subject to differential misclassification). Using prior values of sensitivity and specificity, we randomly select a proportion of exposed and unexposed individuals (within strata of confounders and the outcome) that are reclassified with respect to imidacloprid exposure. The proportion selected for reclassification of exposure is determined by the joint distribution of sensitivity and specificity. These “corrected” data are then used to model the association between imidacloprid exposure and ASD. This process is repeated thousands of times and the results are averaged to obtain the posterior log-odds ratio.

Because the true extent of exposure misclassification was unknown, we estimated posterior ORs under a range of prior misclassification scenarios (i.e. we varied sensitivity from 70 to 100% and false-positive probability from 0 to 20%) in which we assume that misclassification is known with certainty (*certain misclassification* model).

We used 3 levels of priors in our Bayesian analyses: regression parameter priors on the outcome model (*naïve*, *certain misclassification* models), priors for the misclassification model (*certain misclassification* models), and priors on the model that estimates the proportion exposed at each level of the covariates (*certain misclassification* models). MacLehose et al. [22] describe the specifics of these statistical methods in detail.

We report 95% confidence intervals for frequentist models and 95% credible limits for Bayesian models, and to reflect precision we calculated Confidence Limit Ratios (CLR) or Credible Limit Ratios (CrLR), which equal the upper 95% confidence/credible limit divided by the lower 95% limit [41].

All statistical analyses were performed using JAGS version 3.1 and R version 2.9 with the “R2jags” package [42]. For Bayesian models we used Markov-Chain Monte Carlo methods in three chains of 15,000 iterations, including a 2,000 iteration burn-in and assessed convergence using tests recommended by Gelman and colleagues [43]. We calculated posterior ORs and 95% credible intervals using the median and the 2.5th and 97.5th percentiles from the sample of OR estimates.

## Results

### Demographics

The current analysis includes all CHARGE participants with clinical interviews, diagnoses, and interview data completed before September 2011: 587 with confirmed ASD and 356 confirmed to be typically developing (Table 1). Typically developing children were slightly younger (mean 3 y 7 mo vs. 3 y 10 mo for ASD). Mothers of children with ASD had a higher proportion with a college degree (41% vs. 34%) and were less likely than mothers of typically developing children to have weekly or more frequent contact with pets during the prenatal period (46% vs. 55%). Distributions of maternal race/ethnicity and parity and child’s sex were similar between groups. A negligible number of participants had missing information for most covariates.

**Table 1:** Characteristics of study participants in CHARGE through September 2011

	**Autism spectrum disorder**	**Typically developing**
	**N (%)**	**N (%)**
**Imidacloprid usage (ever)**		
No	461 (79)	280 (79)
Yes	115 (20)	75 (21)
Missing	11 (2)	1 (0)
**Imidacloprid usage (prenatal)**		
No	491 (84)	300 (84)
Yes - total	70 (12)	40 (11)
Yes – Consistent†	47 (8)	20 (6)
Yes – Occasional†	15 (3)	14 (4)
Missing	26 (4)	16 (4)
**Maternal education**		
College degree	243 (41)	120 (34)
High school	88 (15)	54 (15)
Some college	256 (44)	182 (51)
Missing	0 (0)	0 (0)
**Child’s age at interview**		
Mean	3.80	3.56
Standard deviation	0.81	0.81
Missing	0	0
**Maternal race/ethnicity**‡		
Asian/Pacific Islander	46 (8)	25 (7)
Black	20 (3)	10 (3)
Multiracial	24 (4)	16 (4)
Native American/Alaskan	3 (1)	1 (0)
Other race	0 (0)	1 (0)
White/Hispanic	140 (24)	69 (19)
White/Non-Hispanic	330 (56)	221 (62)
Missing	24 (4)	13 (4)
**Child’s sex**		
Male	497 (85)	295 (83)
Female	90 (15)	61 (17)
Missing	0 (0)	0 (0)
**Parity**		
Primiparous	160 (27)	99 (28)
2	194 (33)	102 (29)
3	116 (20)	64 (18)
4	59 (10)	50 (14)
>4	51 (9)	38 (11)
Missing	7 (1)	3 (1)
**Pet contact (prenatal)**		
No	168 (29)	97 (27)
Yes	272 (46)	196 (55)
Missing§	147 (25)	63 (18)

† Consistent = reported imidacloprid use for every month of prenatal period; Occasional = reported only some months of use during prenatal period. Frequency is missing if exposure in any month is missing.

‡ Maternal race/ethnicity: single variable combining the two variables: race and ethnicity. Categories were hierarchical: race was assigned first; then among whites, was assigned as Hispanic or non-Hispanic.

§ 213 individuals missing prenatal pet contact information resulting from faulty skip pattern in phone interview software for some participants.

Report of imidacloprid usage was similar between the ASD and typically developing groups (20% vs. 21% for ever use, 12% vs. 11% for prenatal use, Table 1). Mothers reported that they (rather than other household members) applied the insecticide product on the pet in 75% of the families that reported application.

### Imidacloprid-ASD association

**Figure 1:**
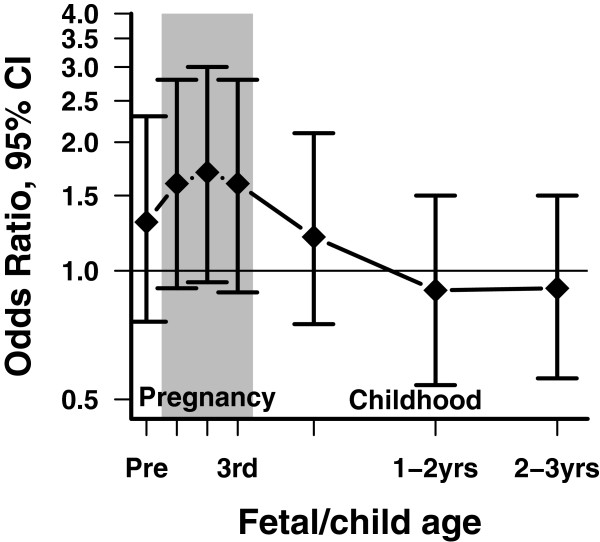
**Adjusted odds ratios and 95% confidence intervals comparing imidacloprid exposure of children with autism spectrum disorder with typically developing controls from the CHARGE data.** Estimates are from separate frequentist, unconditional logistic models for each time period. All models were adjusted for child’s sex, regional center of birth, and age, maternal education, race/ethnicity, and parity and pet ownership during the prenatal period.

Results from the frequentist and *naïve* Bayesian analyses (Table 2), assuming perfect exposure classification, indicated an imprecise, weak positive association between ASD and prenatal imidacloprid exposure compared to typically developing controls: frequentist adjusted OR (95% Confidence intervals [CI]) = 1.3 (0.79-2.2); Bayesian posterior adjusted OR (95% Credible intervals [CrI]) = 1.3 (0.78-2.2). In frequentist models stratified by consistency of use, the OR (95% CI) was 0.69 (0.27-1.8) for occasional users and 2.0 (1.0-3.9) for consistent users. As shown in Figure 1, exposure window analysis indicated that the OR was higher for exposures during the prenatal period than during the first three years of life, though estimates were imprecise. While a model simultaneously adjusting for all exposure periods at once is more desirable than separate models, the high correlation of usage across the exposure windows precludes meaningful inference from such a model.

**Table 2:** Bayesian and frequentist logistic regression results for preferred model comparing the log-odds of imidacloprid exposure during the prenatal period

	**OR**	**(95% CI) †**	**CLR‡**
**Frequentist**			
Crude	1.1	(0.71, 1.6)	2.3
Matching factors only	1.2	(0.79, 1.8)	2.3
Fully adjusted	1.3	(0.79, 2.2)	2.8
Occasional users vs. unexposed§	0.69	(0.27, 1.8)	6.6
Consistent users vs. unexposed§	2.0	(1.0, 3.9)	3.7
**Bayesian**			
Naïve	1.3	(0.78, 2.2)	2.9

† 95% CI – 95% Confidence (frequentist) or Credible (Bayesian) limits

‡ CLR – Confidence or Credible limit ratio = (upper 95% limit/lower 95% limit).

All models were adjusted for child’s sex, regional center of birth, and age, maternal education, race/ethnicity, and parity and pet ownership during the prenatal period. Bayesian priors on regression coefficients and misclassification parameters are described in text.

§ Occasional users: reported imidacloprid use during some, but not all months of prenatal period; Consistent users: reported imidacloprid use during all months of prenatal period.

**Figure 2:**
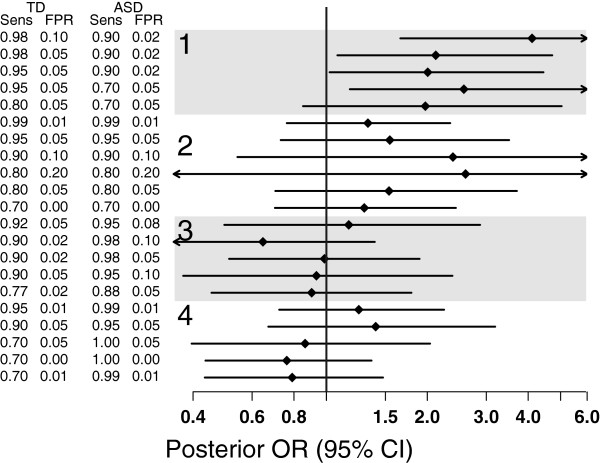
**Adjusted odds ratios and 95% confidence intervals comparing imidacloprid exposure of all children with an autism spectrum disorder (ASD) to that of typically developing (typically developing) controls.** This sensitivity analysis varies sensitivity (Sens) and false positive probability (FPP, 1-specificity) priors used in the Bayesian models assuming known exposure misclassification (*certain misclassification* models). Models are broken into four groups; 1: sensitivity and FPP are greater among controls; 2: sensitivity and FPP are equal between cases and controls (non-differential misclassification); 3: sensitivity and FPP are greater among cases; 4: sensitivity greater among cases, FPP is equal between cases and controls.

We corrected for exposure misclassification under a range of sensitivity and false-positive probability to produce posterior ORs for four distinct groups: 1) sensitivity and false-positive probability assumed greater among controls; 2) non-differential misclassification; 3) sensitivity and false-positive probability assumed greater among cases; 4) sensitivity greater among cases, false-positive probability is equal between cases and controls. Posterior ORs were highest for group 1, i.e., when exposed controls were more likely than exposed cases to report exposure and unexposed controls were more likely to incorrectly report exposure than unexposed cases, while groups 3 and 4, in which exposed cases were more likely than exposed controls to accurately report their exposures, included ORs below one (Figure 2).

#### Sensitivity analyses

Point estimates for a given *certain misclassification* scenario differed little according to the type of regression coefficient prior (informative, vague ~ N (0,10), or tight ~ N (0,1)), except for estimates with the widest confidence intervals (Additional file 1: Figure 1).

## Discussion

In any case–control study, error in exposure measurement is a potential source of bias, and if measurement accuracy differs between cases and controls the direction of bias could be in either direction, depending on the nature of the errors and how they relate to case–control status. We show three examples of ways to potentially mitigate bias from exposure measurement error: 1) restrict potentially exposed individuals to those who likely have most accurate recall (consistent users); 2) examine exposure-outcome associations both within and outside of critical windows of susceptibility to examine measurement error induced by including extraneous exposures; and 3) explicitly correct the OR for potential exposure misclassification under plausible scenarios. We observed that, upon restricting exposure to individuals reporting consistent use of imidacloprid during pregnancy, the odds of reported imidacloprid exposure among mothers of children with ASD is twice that of mothers of TD children. Figure 1 shows that we also observed an increase in the OR during pregnancy versus early postnatal exposures, consistent with reports of other pesticide-ASD associations. Over a range of potential misclassification scenarios, we observed posterior ORs ranging from slightly decreased risk (OR ~ 0.6) to strongly increased risk (OR ~ 4.0) for ASD diagnosis with prenatal imidacloprid exposure. To our knowledge, no previous authors have reported on associations between imidacloprid and developmental outcomes in humans and, while bias cannot be completely ruled out, we believe that the current analysis presents a possible association that warrants further epidemiologic and biologic investigation.

Imidacloprid was first registered for use as a pesticide in the US in 1994 and is widely used by pet owners, yet little work has since been conducted to assess human health effects. Initial tests for developmental toxicity of imidacloprid indicated oral dosing of pregnant rats to be associated with decreased motor activity and decreased caudate/putamen thickness in offspring (female rats only) [17]. Abou-Donia et al. noted that prenatally exposed rats displayed sensorimotor deficits and increased expression of glial fibrillary acidic protein, which has been previously reported among persons with ASD [44,45], and in a mouse model for ASD [46]. Further, the primary action of imidacloprid on nicotinic-cholinergic receptors is similar to that of organophosphates [47], which have previously been associated with ASD [14,15]. Dermal absorption of imidacloprid can result from petting recently treated animals [48], but the dose that could potentially reach the fetus is unknown [49], and no known studies have examined potential neurotoxic effects of imidacloprid on the human fetus. We did not have data on how much physical contact the mother had with the pet that was treated.

Case control studies utilizing self-report to identify exposures of interest are prone to differential exposure misclassification that can lead to bias. The CHARGE Study utilizes maternal recall of household pesticide use from, on average, 4 years in the past; independent assessment of household pesticide exposures was not feasible. Bayesian methods to correct for exposure misclassification can sometimes circumvent these shortcomings, but the results are sensitive to assumptions about the magnitude and precision of misclassification of the exposure.

As shown in Table 2, adjusted frequentist models and *naïve* Bayesian models agree that there is no appreciable difference in exposure between children with ASD and TD children, providing remarkably similar point estimates and precision. An elevated risk associated with exposure is, nevertheless, suggested by the susceptibility window analysis in Figure 1, and the doubling of odds for consistent users of imidacloprid-containing pet products. In addition to signifying a possible etiologic relationship, this elevation in reported imidacloprid use during pregnancy is also consistent with a) a mitigating factors such as small sample size or bias due to confounding or b) recall bias arising from (for example) concerns about prenatal exposures in which improved reporting or over-reporting of exposure among mothers of children with ASD is high in the prenatal period but is lower in early life.

Any interpretation of our results must be tempered by the following caveats: a) our initial assumptions about how the magnitude of misclassification differs between case and control mothers may not be plausible (which warrants estimation of the OR under multiple scenarios) b) the apparently higher ORs for exposure during pregnancy or among consistent users could also be consistent with recall bias if sensitivity and specificity of recall varied over time or if mothers of children with ASD disproportionately report consistent (rather than occasional) use, and c) imprecision in the posterior ORs indicates that they are derived from relatively small numbers of individuals in some strata. Because of these caveats, caution is warranted in interpreting our elevated ORs as indicative of a true association – other studies could build on our results by estimating misclassification parameters across time, by prospectively assessing exposure, or by focusing on populations with a higher proportion of households using this pesticide (such as pet owners).

The analysis shown in Figure 2 indicates that, in *misclassification* models, small changes in the false-positive probability estimate for children with ASD can lead to disproportionately large changes in the posterior OR, which has also been observed by Marshall [50] and Gustafson and colleagues [21]. *A priori*, one might expect that sensitivity and false-positive probability would both be higher among mothers of children with ASD (group 3 in Figure 2), resulting in upward bias. Higher reporting of exposure among mothers of TD children (and the highest observed ORs - group 1) seems most implausible. Our susceptibility window analysis and the analysis in which exposure is stratified by consistency of reported use suggest the possibility that exposure misclassification could be obscuring, rather than enhancing, an association between ASD and imicloprid. If this were the case, the summary OR of the adjusted frequentist model and the naïve Bayesian analysis may be a) biased because of exposure misclassification due to inaccurate reporting or b) a poor estimate of an etiologic relationship due either to a classification scheme in which potentially biologically relevant exposure (i.e. high exposed groups or exposure during neurogenesis) is lumped with less relevant exposure. An analysis by Roberts et al. suggests that there may be a critical window in early pregnancy during which exposure to organophosphate pesticides may magnify risk for development of ASD [14]. The trade-off of focusing on subgroups or specific windows of exposure is a loss of precision in estimating an association; hence, we used multiple approaches to address exposure misclassification.

Our findings underscore the need for validation studies of maternal self-report of household exposures in studies of childhood behavioral and neurologic disorders. Because we were unable to place informative central estimates on sensitivity and specificity of imidacloprid report during the prenatal period, we can provide only posterior ORs under a range of plausible estimates. If the posterior ORs in Figure 2 indicated a consistent direction of association between imidacloprid and ASD, there would be little concern that inaccurate recall of exposure could be masking a true association. Unfortunately, such validation studies have yet to be conducted. One area in which such studies have been conducted is in regard to prenatal cigarette smoking and birth defects, likely due to the ready availability of a valid biomarker of exposure (cotinine) and the short follow-up time necessary to evaluate health outcomes in newborns. MacLehose et al. [22] applied Bayesian methods for exposure misclassification to an analysis of maternal smoking and orofacial clefts. The authors observed a 20% increase in the posterior OR for the prenatal smoking-cleft lip/palate association after correcting for misclassification and allowing for sampling error in the sensitivity and false-positive probability estimates. In a study of smoking and invasive pneumococcal disease, Chu et al. [51] applied a Bayesian analysis under a range of sensitivity and false-positive probability values similar to ours, though the authors allowed for both for uncertainty in the misclassification parameters as well as correlation between sensitivity and false-positive probability, which we did not do. In the case of both MacLehose et al. and Chu et al., the authors had a range of prior validity studies with validated biomarkers to inform the sensitivity analysis and potentially allow estimation of a central, posterior OR. Were that information available to us, our 95% posterior credible intervals could more accurately account for uncertainty about sensitivity and false-positive probability. Bayesian analysis allows explicit incorporation of prior information, which is a relative strength compared to our other methods of dealing with exposure misclassification.

While biomarkers have been developed for maternal exposures to certain pesticides, much work remains to be done in this field [5], and the delay between birth and the diagnosis of ASD is problematic both for maternal recall as well as for establishing correlations between biomarker levels after diagnosis with prenatal exposures. One potential pitfall is that a given biomarker may not necessarily isolate a single exposure, since several pesticides may result in indistinguishable metabolic byproducts [5]. In our analysis of CHARGE data, adjustment for exposure to pyrethroids (~31% reported use), another household pesticide often used in flea and tick products for pets, changed the OR by less than 5%, as did restricting our analysis to pet owners (not shown).

Because imidacloprid effects on human development are not well understood, we were unable to place an informative prior estimate on the OR for imidacloprid exposure. Consequently, the variation in our *misclassification* models likely overestimates the effect that slight changes in sensitivity and false-positive probability estimates would have on the posterior OR for exposures with extensive prior literature. For example, parental autoimmune disease associations with ASD have been well-studied [52-55], so a posterior OR for autoimmune disease-ASD associations would show less variation across sensitivity and false-positive probability estimates than is observed in the current study due to an informative prior. Thus, the variation in ORs across misclassification parameters (Figure 2) would be smaller in association studies of more well-researched risk factors in autism. Additionally, our lack of informative prior on the OR for imidacloprid exposure also contributed to the similarity between the naïve Bayesian analysis and the frequentist analysis, and, in this case, the advantages of the naïve Bayesian over the frequentist analysis are mainly theoretical. The difference would have been more pronounced had we either a) been able to provide and informative prior on the OR for imidacloprid use or b) included some confounders that result in empty strata, in which case the frequentist model may not have converged or would have reduced precision of the OR estimate.

We considered the potential for recently reported variability in ASD risk associated with season and vitamin use to impact our results [56-58]. Imidacloprid usage patterns did not vary by season and in frequentist logistic models with additional adjustment for winter birth (January-March) the log-odds ratio for imidacloprid exposures (consistent and occasional use) changed by less than 1% (not shown). Adjustment for prenatal vitamin use changed the log-odds ratio by less than 3% (not shown).

Little is known about the causes of ASD, so uncontrolled confounding or selection bias cannot be ruled out [59]. The CHARGE study collected vast amounts of information on potential covariates from multiple sources, allowing for a potential reduction in confounding or selection bias that may be present in larger, records-based studies that cannot collect detailed data at the individual level. In spite of the stated shortcomings, the current analysis represents a thorough examination of the association between a household pesticide and subsequent ASD diagnosis that addresses potentially differential misclassification of exposure, thereby overcoming a major challenge in case–control studies when persistent biomarkers for exposure have not been identified.

Ultimately, we do not currently know the true distribution of imidacloprid exposure in the CHARGE study, and we also do not know how closely applications of flea and tick products to household pets correlates with fetal exposure. We did not address imidacloprid exposure from food or from residential proximity to commercial spraying, nor did we distinguish genetically susceptible case subgroups, who may have different associations with environmental exposures.

## Conclusions

Our findings highlight the need for validation studies of exposures in different time intervals. Future large cohorts (e.g. the National Children’s Study [60]) have an opportunity to improve on the exposure misclassification estimates by collecting both prospective and retrospective information on pesticide exposures. Analyses similar to ours will benefit from reporting of sensitivity and specificity estimates of household product use based on comparisons of retrospective reporting relative to prenatal reporting or biomarker measurements that accurately reflect prenatal exposures. Links between imidacloprid and developmental outcomes in animal studies, the potential for exposure from household or nearby agriculture applications, and the results shown in frequentist analyses and in Bayesian models with misclassification-corrected models for some scenarios provide hints that this association warrants further study.

## Abbreviations

ASD: Autism spectrum disorder; CHARGE: Childhood autism risks from genetics and environment; CI: Confidence interval; CLR: Confidence limit ratio; CrI: Credible interval; CrLR: Credible limit ratio; DAG: Directed acyclic graph; JAGS: Just another gibbs sampler; MIND: Medical investigation of neurodevelopmental disorders; OR: Odds ratio.

## Competing interests

The authors declare no competing interests.

## Authors’ contributions

AK conducted the literature review and was responsible for drafting the primary manuscript. IH-P was responsible for design of the CHARGE study, obtaining funding, and directing its implementation. All authors contributed substantially to revision of subsequent versions of the manuscript. All authors read and approved the final manuscript.

## Supplementary Material

Additional file 1 Autism and Imidacloprid. Click here for file
