# Supplementary material for: Autism spectrum disorder, flea and tick medication, and adjustments for exposure misclassification: the CHARGE (CHildhood Autism Risks from Genetics and Environment) case–control study
Source: Environ Health. 2014 Jan 23;13:3. doi: 10.1186/1476-069X-13-3 (PMC3922790; doi:10.1186/1476-069X-13-3)
Supplement: Additional file 1 — Autism and Imidacloprid. [file 1476-069X-13-3-S1.pdf]

Sensitivity analyses

Methods

As MacLehose et al.[1] point out, the posterior OR can be highly sensitive to misclassification parameter priors since the data contain very little information on misclassification. We performed additional analyses using varied estimates of Sens and FPR. We used the *certain misclassification* model as a template for these analyses to estimate the OR of imidacloprid exposure at several, fixed estimates of misclassification. For the overall ASD-TD model, we varied Sens from 50% to 99% and FPR from 0 to 10%. Additionally, we examined the influence of our priors on regression parameters by examining posterior estimates derived using vague (~N (0,10), tight (~N (0,1)), and informative regression parameter priors. Note that models with informative priors are duplicated from the figure in the main text.

Results

In general, point estimates for a given *certain misclassification* scenario differed little according to the type of regression coefficient prior (informative, vague ~ N (0,10), or tight ~ N (0,1)), except for estimates with the widest confidence intervals (Figure 2). Sensitivity analysis ORs broke generally into 4 distinct groups: 1) Sens and FPR are greater among controls; 2) Sens and FPR are equal between cases and controls (non-differential misclassification); 3) Sens and FPR are greater among cases; 4) Sens greater among cases, FPR is equal between cases and controls. Posterior ORs were highest for group 1, while groups 3 and 4 included some ORs below the null.

Discussion

The sensitivity analysis shown in figure 2 indicates that, in *certain misclassification* models, small changes in the FPR estimate for children with ASD can lead to disproportionately large changes in the posterior OR, while regression coefficient priors have relatively little effect. Marshall[2] and Gustafson et al.[3] showed that small

changes in misclassification parameters can produce large changes in posterior ORs. Additionally, the sensitivity analysis shown in Online appendix table 1 indicates that the posterior OR depends on the precision (certainty) of the misclassification priors, particularly for TD children.

Online appendix Table 1: Table 1: Informative prior means and 95% credible intervals for regression coefficients in outcome model based on estimates from prior literature or N(0,10) vague priors*.

	**Regression priors**	**(95% CrI)**
**Intercept**	0.0	(-6.2, 6.2)
**Imidacloprid**	0.0	(-6.2, 6.2)
**Matching factors (Regional center, child's sex, child's age)**	0.0	(-6.2, 6.2)
**Maternal education (some college)**	-0.24	(-0.32, -0.15)
**Maternal education (high school)**	-1.0	(-1.1, -0.89)
**Race**	-0.14	(-0.30, 0.015)
**Parity (linear)**	-0.18	(-0.26, -0.089)

*Vague prior specification was used for matching factors and regression coefficients for which no adequate prior information existed. The vague prior corresponds to prior OR (95%CrI) of 1.00 (0.002, 492).

See variable descriptions in the main text

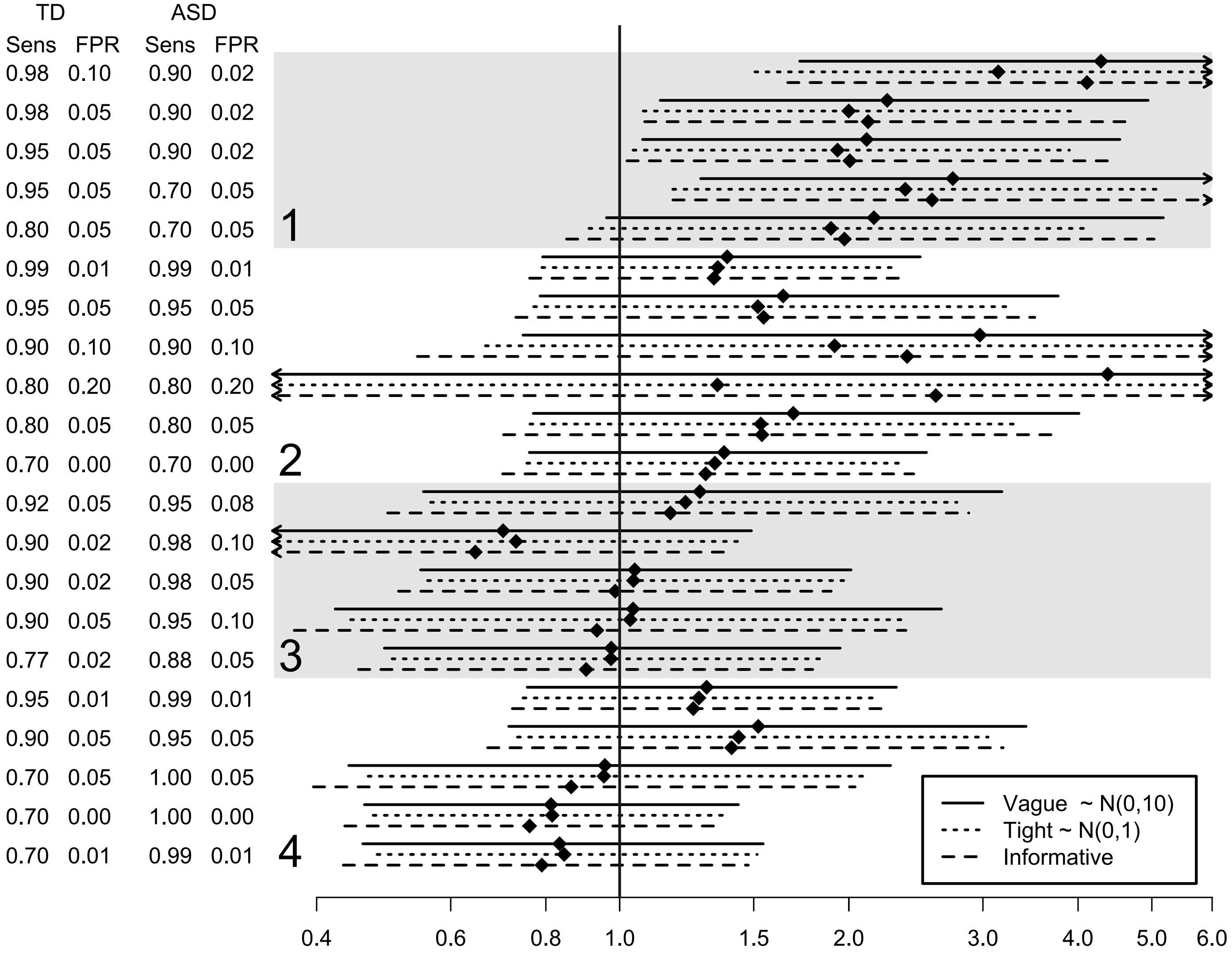

Online appendix Figure 1: Adjusted odds ratios and 95% confidence intervals comparing imidacloprid exposure of all children with an autism spectrum disorder (ASD) to that of typically developing (TD) controls. This sensitivity analysis varies the use of regression coefficient priors (Vague, Tight, Informative) and estimates of Sensitivity (Sens) and False positive rate (FPR) used in the Bayesian models assuming known exposure misclassification (*certain misclassification* models). Models are broken into four groups; 1: Sens and FPR are greater among controls; 2: Sens and FPR are equal between cases and controls (non-differential misclassification); 3: Sens and FPR are greater among cases; 4: Sens greater among cases, FPR is equal between cases and controls.

Online Appendix References

1. MacLehose RF, Olshan AF, Herring AH, Honein MA, Shaw GM, and Romitti PA. Bayesian methods for correcting misclassification: an example from birth defects epidemiology. *Epidemiology*. 2009; 20:27--35.

2. Marshall JR. The use of dual or multiple reports in epidemiologic studies. *Statistics in medicine*. 1989; 8:1041--1049.

3. Gustafson P, Le ND, and Saskin R. Case-control analysis with partial knowledge of exposure misclassification probabilities. *Biometrics*. 2001; :598-609.
